# Supplementary material for: A set of multi-entry identification keys to African frugivorous flies (Diptera, Tephritidae)
Source: Zookeys. 2014 Jul 24;(428):97–108. doi: 10.3897/zookeys.428.7366 (PMC4143993; doi:10.3897/zookeys.428.7366)
Supplement: Supplementary material 4 — Key to Capparimyia [file zookeys-428-097-s004.zip › SF4_ZooKeys_key to Capparimyia/lucid_player/help/help/opening_a_key.htm]

Opening a Key


# Opening a Key

When using the Application Player, open a key by choosing *Open*�
from the *Key* menu or clicking
on the *Open Key* button  on the toolbar.

Browse for the key in the *Open Key*
dialog box.

The *Key* menu maintains a list
of the most recently opened keys, providing a shortcut for opening these
keys.

*Open Key* and the *Recently
Opened Keys* list are not available in the Applet Player.

**Note:** The first time
the Lucid Player loads you will be asked to define a workspace folder.
The workspace folder is where your preferences for the Lucid Player are
stored. The Player must have read and write access to this folder.
